# C16, a PKR inhibitor, suppresses cell proliferation by regulating the cell cycle via p21 in colorectal cancer

**DOI:** 10.1038/s41598-024-59671-7

**Published:** 2024-04-19

**Authors:** Yu Hashimoto, Yoshio Tokumoto, Takao Watanabe, Yusuke Ogi, Hiroki Sugishita, Satoshi Akita, Kazuki Niida, Mirai Hayashi, Masaya Okada, Kana Shiraishi, Kazuhiro Tange, Hideomi Tomida, Yasunori Yamamoto, Eiji Takeshita, Yoshio Ikeda, Taro Oshikiri, Yoichi Hiasa

**Affiliations:** 1 Department of Gastroenterology and Metabology, Ehime University Graduate School of Medicine, Shitsukawa, Toon, Ehime 791-0295 Japan; 2 Department of Gastrointestinal Surgery and Surgical Oncology, Ehime University Graduate School of Medicine, Toon, Ehime 791-0295 Japan; 3 Department of Minimally Invasive Gastroenterology, Ehime University Graduate School of Medicine, Shitsukawa, Toon, Ehime 791-0295 Japan; 4 Department of Inflammatory Bowel Diseases and Therapeutics, Ehime University Graduate School of Medicine, Shitsukawa, Toon, Ehime 791-0295 Japan; 5 Endoscopy Center, Ehime University Hospital, Shitsukawa, Toon, Ehime 791-0295 Japan

**Keywords:** Colorectal cancer, C16, Cell cycle, Tumor growth, Cancer, Drug discovery, Gastroenterology, Molecular medicine, Oncology

## Abstract

Double-stranded RNA-activated protein kinase R (PKR) is highly expressed in colorectal cancer (CRC). However, the role of PKR in CRC remains unclear. The aim of this study was to clarify whether C16 (a PKR inhibitor) exhibits antitumor effects and to identify its target pathway in CRC. We evaluated the effects of C16 on CRC cell lines using the MTS assay. Enrichment analysis was performed to identify the target pathway of C16. The cell cycle was analyzed using flow cytometry. Finally, we used immunohistochemistry to examine human CRC specimens. C16 suppressed the proliferation of CRC cells. Gene Ontology (GO) analysis revealed that the cell cycle-related GO category was substantially enriched in CRC cells treated with C16. C16 treatment resulted in G1 arrest and increased p21 protein and mRNA expression. Moreover, p21 expression was associated with CRC development as observed using immunohistochemical analysis of human CRC tissues. C16 upregulates p21 expression in CRC cells to regulate cell cycle and suppress tumor growth. Thus, PKR inhibitors may serve as a new treatment option for patients with CRC.

## Introduction

Colorectal cancer (CRC) is the third most common cancer and the second most common cause of cancer-related deaths worldwide^1^. The incidence of CRC is rapidly increasing in several countries, likely owing to changes in lifestyle factors and diet. Definite risk factors for CRC include the consumption of red and processed meat, obesity, and physical inactivity^2–5^. Colorectal cancer is expected to remain a common cancer in society owing to population aging and growth. The number of older adults with cancer is predicted to double worldwide by 2035, with CRC being one of the main contributors^6^. The prognosis of patients with advanced stage CRC remains poor despite advances in molecularly targeted agents and chemotherapy for CRC. Therefore, new targets for CRC treatment are needed.

The multistep genetic model of colorectal carcinogenesis is widely accepted as a paradigm of solid tumor progression. According to this model, APC inactivation occurs first; this is followed by oncogenic KRAS mutations in the adenomatous stage. Eventually, inactivation of the tumor suppressor gene *TP53* on chromosome 17p occurs during the transition to malignancy^7^. TP53 regulates the expression of genes involved in cell cycle, including p21 (CDKN1A). p21 is a cyclin-dependent kinase inhibitor, and its decreased expression correlates with metastasis development and poor patient survival^8–11^.

Double-stranded RNA-dependent protein kinase (PKR) is a serine/threonine protein kinase that is expressed throughout the body. It plays an important role in the antiviral and anti-proliferative effects of interferon^12^ and is overexpressed and activated in several malignant diseases^13–15^. Analysis of colon cancer specimens has shown that PKR interacts with major tumor suppressor genes such as p53 and plays an essential role in tumor suppressor functions^16^. In contrast, malignant transformation from normal mucosa to adenoma or adenocarcinoma coincides with an increase in PKR expression^17^. The role of PKR in CRC remains unclear.

The PKR inhibitor oxindole/imidazole (C16) is an ATP-binding site-directed small molecule that inhibits PKR autophosphorylation^18^. Our previous study showed that C16 suppresses tumor progression in hepatocellular carcinoma (HCC)^19^. Interestingly, the Cancer Genome Atlas database shows that the expression of PKR in CRC is higher than that in HCC, suggesting that C16 may be more effective in CRC. Therefore, in this study, we aimed to investigate the therapeutic effects of C16 on CRC cell growth and tumor progression.

## Results

### PKR induces the proliferation of CRC cells

**Figure 1:**
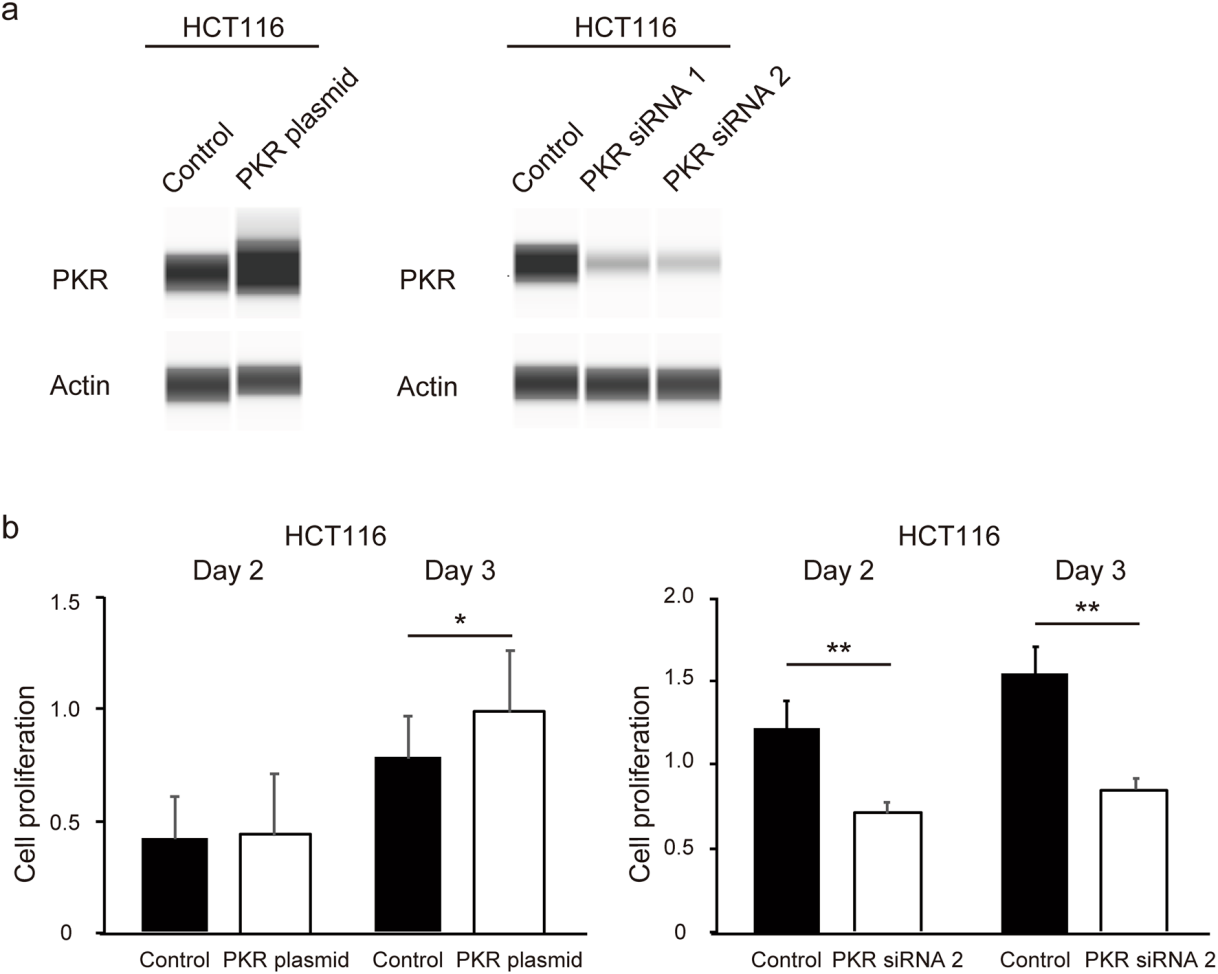
PKR induces the proliferation of CRC cells. HCT116 cells were seeded in a six-well flat-bottomed plate and transfected with PKR plasmid and PKR siRNA1 or siRNA2 for 48 h; thereafter, proteins were extracted and analyzed. The expression of PKR in HCT116 was determined using western blotting. The original gel images of western blotting are shown in Supplemental Fig. S1 (**a**). To investigate the effects on CRC cell proliferation in vitro with the MTS assay, CRC cells were seeded in a 96-well flat-bottomed plate with the PKR plasmid and PKR siRNA. Data are shown as mean ± standard error of six replicates. *p < 0.05, **p < 0.01 compared to the group with control plasmid and control siRNA using Mann–Whitney U test (**b**). *PKR* protein kinase R, *CRC* colorectal cancer.

Western blot analysis was performed to identify changes in PKR expression. We transfected PKR plasmid and *PKR* siRNA into HCT116 cells. We confirmed that the expression of PKR was upregulated by PKR plasmid and knocked down by *PKR* siRNA (Fig. 1a). We then performed the MTS assay to assess the proliferation of HCT116 cells (a p53 wild-type CRC cell line) transfected with PKR plasmid and *PKR* siRNA. The PKR plasmid increased the proliferation and *PKR* siRNA1 and siRNA2 decreased the proliferation of CRC cells (Fig. 1b).

### C16 suppresses the proliferation of CRC cells in a dose-dependent manner in vitro

**Figure 2:**
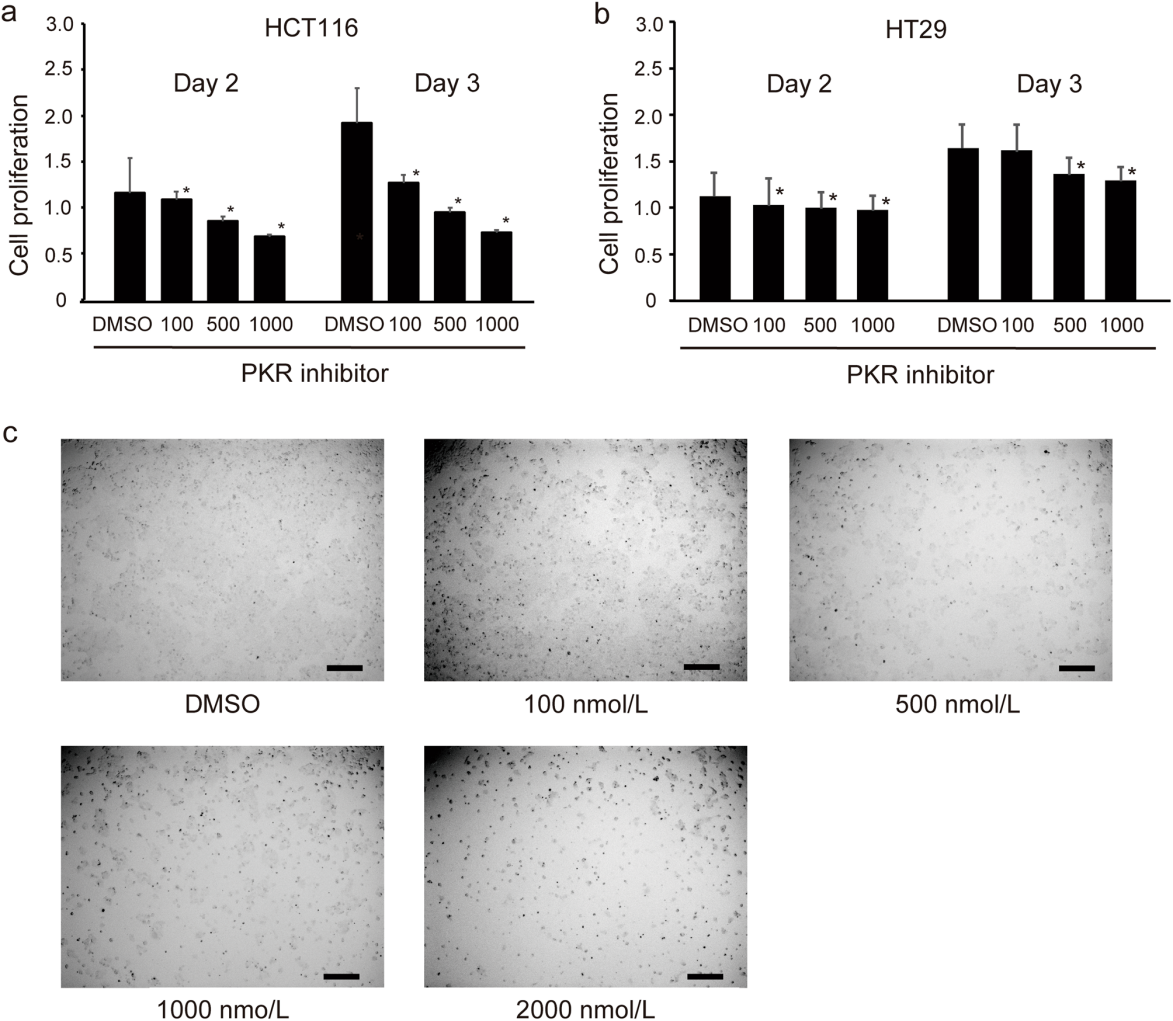
Treatment with C16 PKR inhibitor suppresses CRC cell proliferation in a dose-dependent manner in vitro. (**a**) HCT116 cells, (**b**) HT29 cells. Data are shown as mean ± standard error of six replicates. *p < 0.05, **p < 0.01 compared to the group treated with DMSO (Mann–Whitney U test). (**c**) HCT116 cells were seeded in a six-well plate, cultured at 37 °C for 24 h, and treated with C16 at 100, 500, 1000, and 2000 nM. The microscopic photographs of the wells with C16 at different concentrations. Scale bar, 200 µm. *DMSO* dimethyl sulfoxide, *PKR* protein kinase R, *CRC* colorectal cancer.

The MTS assay was performed to assess the proliferation of CRC cells treated with 0, 100, 500, and 1000 nM C16. The division of HCT116 cells decreased following C16 treatment in a dose-dependent manner (Fig. 2a). The suppressive effect of C16 treatment on CRC cell proliferation was also observed in another p53 mutant CRC cell line, HT29 (Fig. 2b). No morphological changes were observed in the cells 24 h after C16 exposure, suggesting that this inhibitor does not exert substantial toxic effects (Fig. 2c).

### C16 regulates the expression of cell cycle-related genes in CRC cells

**Figure 3:**
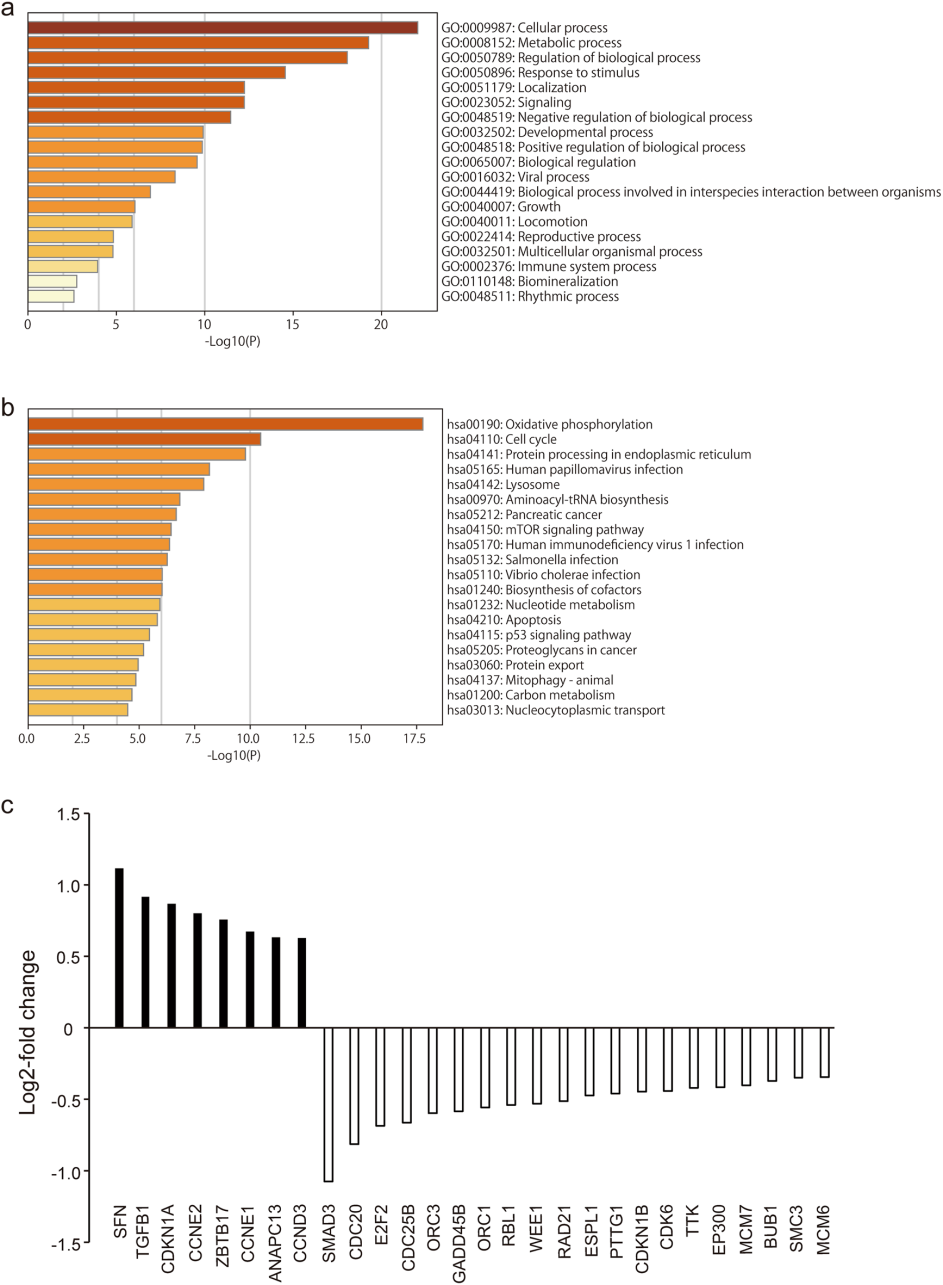
C16 regulates the expression of cell cycle-related genes in CRC cells. Functional enrichment analysis was performed using Metascape and DAVID. RNA-Seq analysis was performed using RNA extracted from HCT116 cells 24 h after treatment with C16 diluted to 500 nM or DMSO (control). Functional enrichment results for GO (**a)** and KEGG (**b**) pathways, and a heatmap showing the top 19 and 20 clusters colored according to the p-value (a darker color indicates a lower p-value). (**c**) RNA-Seq analysis of cell cycle-related gene expression. The y-axis shows the ratio of the average value of fragments per kilobase of transcript per million mapped reads (FPKM) of three HCT116 cells treated with C16 to the average value of cells treated with DMSO. *DMSO* dimethyl sulfoxide, *PKR* protein kinase R, *CRC* colorectal cancer, *GO* Gene Ontology, *KEGG* Kyoto Encyclopedia of Genes and Genomes.

RNA-sequencing (RNA-Seq) analysis of HCT116 cells revealed 1542 potential target genes whose expression is regulated by C16. Comprehensive gene expression analysis using Metascape and DAVID showed that cellular processes had the highest enrichment in Gene Ontology (GO) analysis and the cell cycle-related gene group was the second highest enriched group according to analysis of Kyoto Encyclopedia of Genes and Genomes (KEGG) pathway (Fig. 3a, b). Among cell cycle-related genes, the expression of eight genes including p21 (*CDKN1A*) and TGF-β1 (*TGFB1*) was upregulated after C16 exposure (Fig. 3c, Supplemental Table 1). These results indicate that C16 regulates the cell cycle pathway by upregulating the expression of cell cycle-related genes such as *CDKN1A*.

**Table 1:** Relationship between p21 expression and clinicopathological features of patients with colorectal cancer (CRC).

Variable	P21 expression (n = 22)		
	Low (%)	High (%)	p^a^
All patients	12 (100)	10 (100)	
Age	72.3 ± 7.7	68.1 ± 6.5	0.205
Sex, male	5 (42)	5 (50)	0.696
Location, right	3 (25)	4 (40)	0.656
Tumor diameter (cm)	4.8 ± 2.3	3.0 ± 1.4	0.048
TNM stage			0.043
I/II	4 (33)	8 (80)	
III/IV	8 (67)	2 (20)	
Depth of invasion			0.074
T1/T2	2 (17)	6 (60)	
T3/T4	10 (83)	4 (40)	
Lymph node metastasis			0.099
N0	5 (42)	8 (80)	
N1/N2/N3	7 (58)	2 (20)	
Distant metastasis, M1	3 (25)	0	0.221
Differentiation, poor	4 (33)	1 (10)	0.323

^a^Two-sided Fisher’s exact test.

### C16 regulates CRC cell cycle

**Figure 4:**
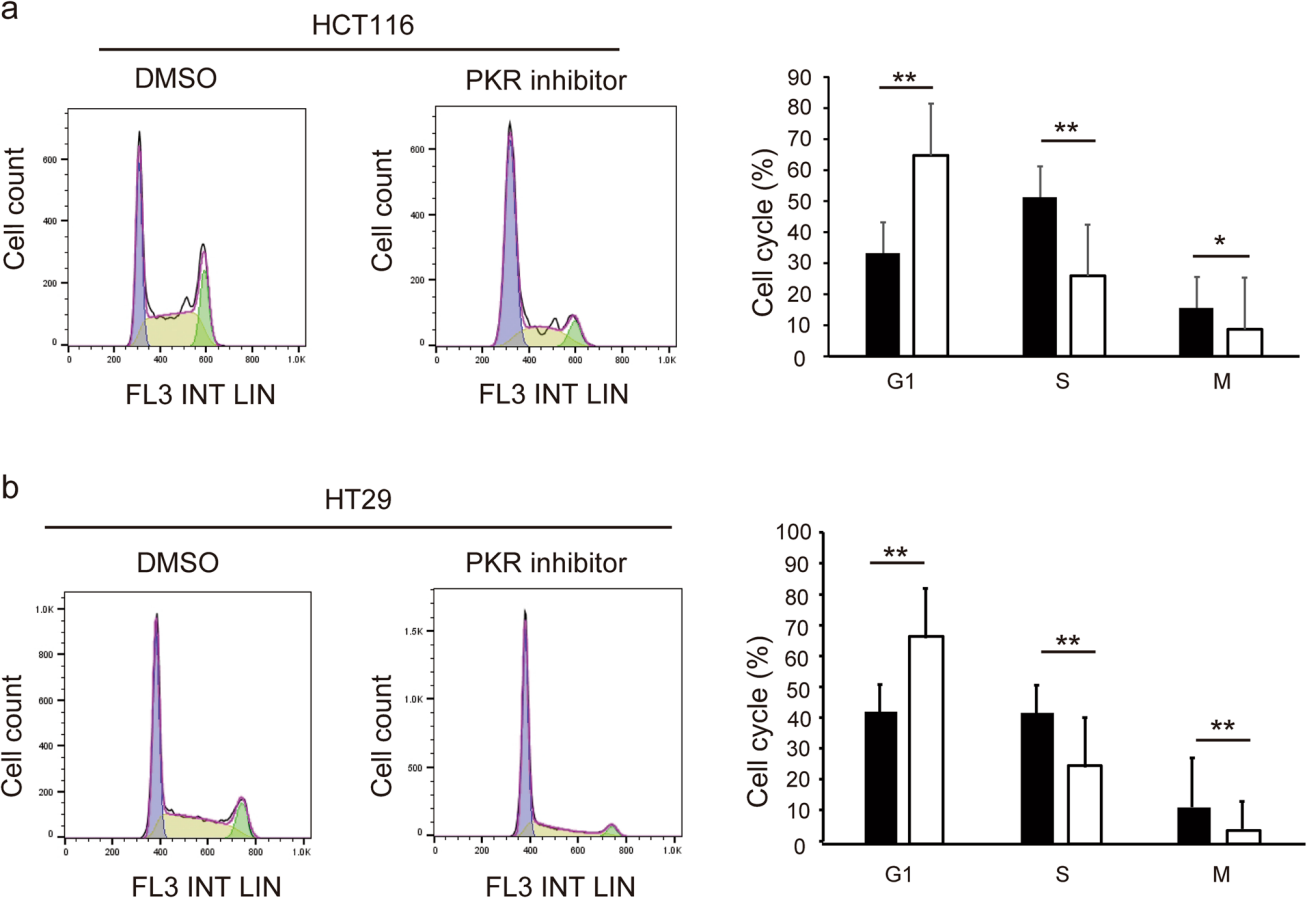
C16 regulates the cell cycle of CRC cells. Flow cytometry analysis was performed after treatment with C16 diluted to 500 nM. Two experimental replicates of cell cycle phase distribution were quantified. The number of cells in the G1 phase increased, whereas the number of cells in the S and G2 phases decreased. (**a**) HCT116 cells; (**b**) HT29 cells. **p < 0.01, *p < 0.05 compared to the group without C16 treatment (Mann–Whitney U test). *DMSO* dimethyl sulfoxide, *PKR* protein kinase R, *CRC* colorectal cancer.

Flow cytometry showed that C16 regulates CRC cell cycle. Following treatment with C16, the number of HCT116 cells in the G1 phase increased and the number of cells in the S and M phases decreased (Fig. 4a). The regulatory effects of C16 treatment on CRC cells were also observed in HT29 cells (Fig. 4b). These results demonstrate that C16 regulates the cell cycle by inhibiting the transition from the G1 to S phase. In addition, apoptosis assays using flow cytometry showed that the rate of apoptosis clearly increased after 48 h C16 treatment. The percentage of annexin V-positive cells defined as apoptotic cells was as follows: control DMSO, 10.5%; C16, 19.4% in HCT116 cells (Supplemental Fig. S2).

### C16 increases p21 expression in CRC cells

**Figure 5:**
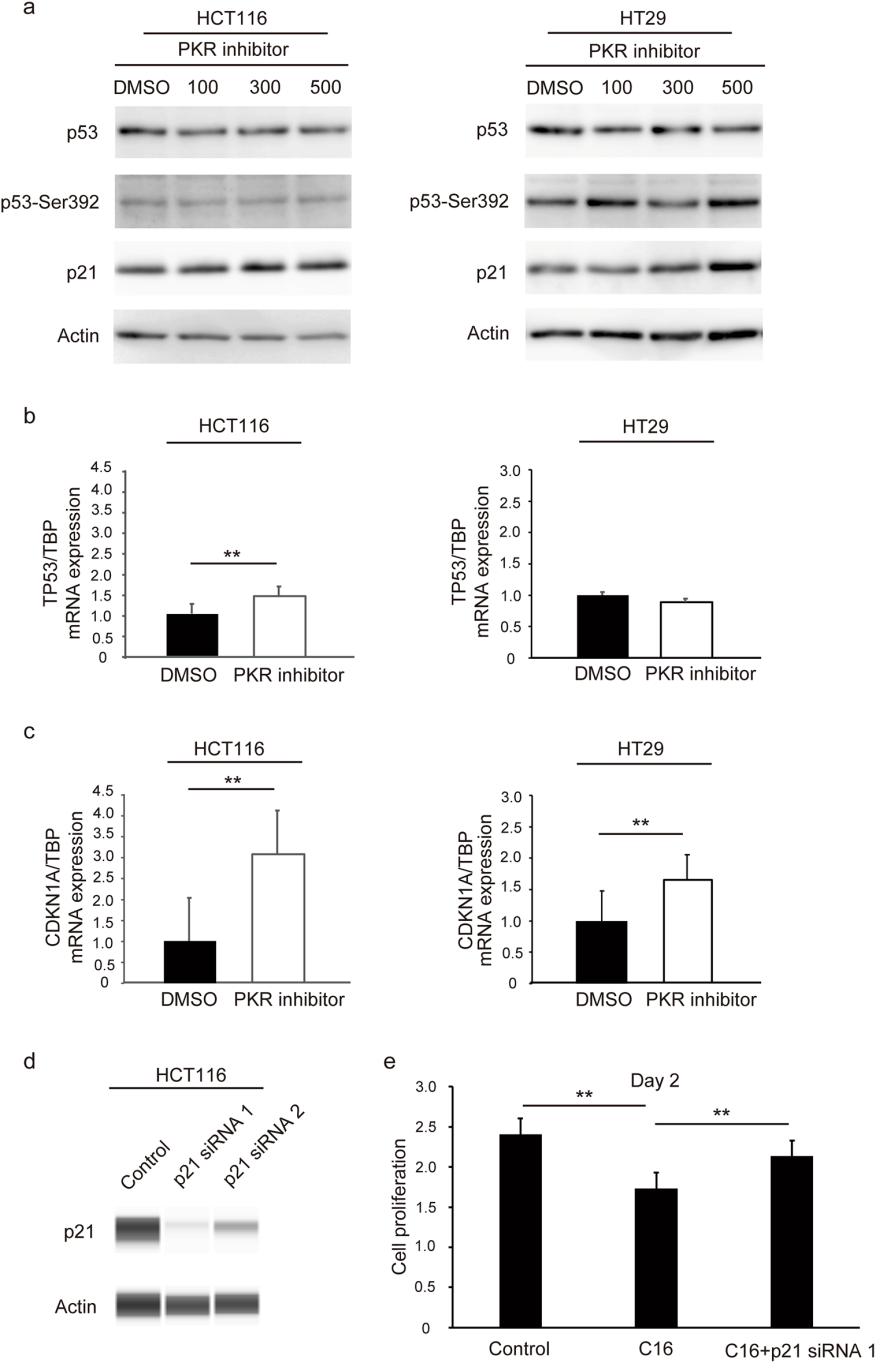
C16 increases the expression of p21 in CRC cells. CRC cells were treated with 100, 300, and 500 nM C16 for 24 h. DMSO was used as a control. The expression of p53, phosphorylated p53, and p21 was determined using western blotting in HCT116 and HT29 cells. The original gel images of western blotting are shown in Supplemental Fig. S3 (**a**). The expression of *TP53* and *CDKN1A* mRNA was measured using real-time PCR. (**b**) TP53, (**c**) CDKN1A. Data are shown as mean ± standard error of six replicates. **p < 0.01 compared to the group without C16 treatment (Mann–Whitney U test). HCT116 cells were seeded in a six-well flat-bottomed plate and transfected with p21 siRNA1 or siRNA2 for 48 h treatment, and then proteins were extracted and analyzed. The expression of p21 was determined using western blotting in HCT116. The original gel images of western blotting are shown in Supplemental Fig. S1 (**d**). To investigate the effects on CRC cell proliferation in vitro with the MTS assay, CRC cells were seeded in a 96-well flat-bottomed plate with C16 and p21 siRNA. Data are shown as mean ± standard error of six replicates. **p < 0.01 compared to the control group (DMSO) with Mann–Whitney U test (**e**). *DMSO* dimethyl sulfoxide, *PKR* protein kinase R, *CRC* colorectal cancer.

We performed western blotting and real-time reverse transcription-polymerase chain reaction (RT-PCR) experiments to examine the effects of C16 on p21 expression after exposing HCT116 cells to 0, 100, 300, or 500 nM of C16. C16 increased p21 and p53 protein expression in HCT116 and HT29 cells (Fig. 5a). Furthermore, it increased *CDKN1A* and *TP53* mRNA expression in HCT116 cells (Fig. 5b) and *CDKN1A* expression in HT29 cells (Fig. 5c). These results indicate that C16 regulates p21 expression. We also performed western blotting and MTS assay to confirm that p21 siRNA reverses the tumor growth-suppressive effects of C16. p21 protein expression was knocked down by p21 siRNA1 and siRNA2 (Fig. 5d). We subsequently transfected p21 siRNA to HCT116 cells treated with C16. The MTS assay showed that p21 siRNA reverses the tumor growth-suppressive effects of C16 (Fig. 5e). These results indicate that the tumor growth-suppressive effects of C16 is dependent on p21.

### p21 expression decreases in advanced human CRC tissues

**Figure 6:**
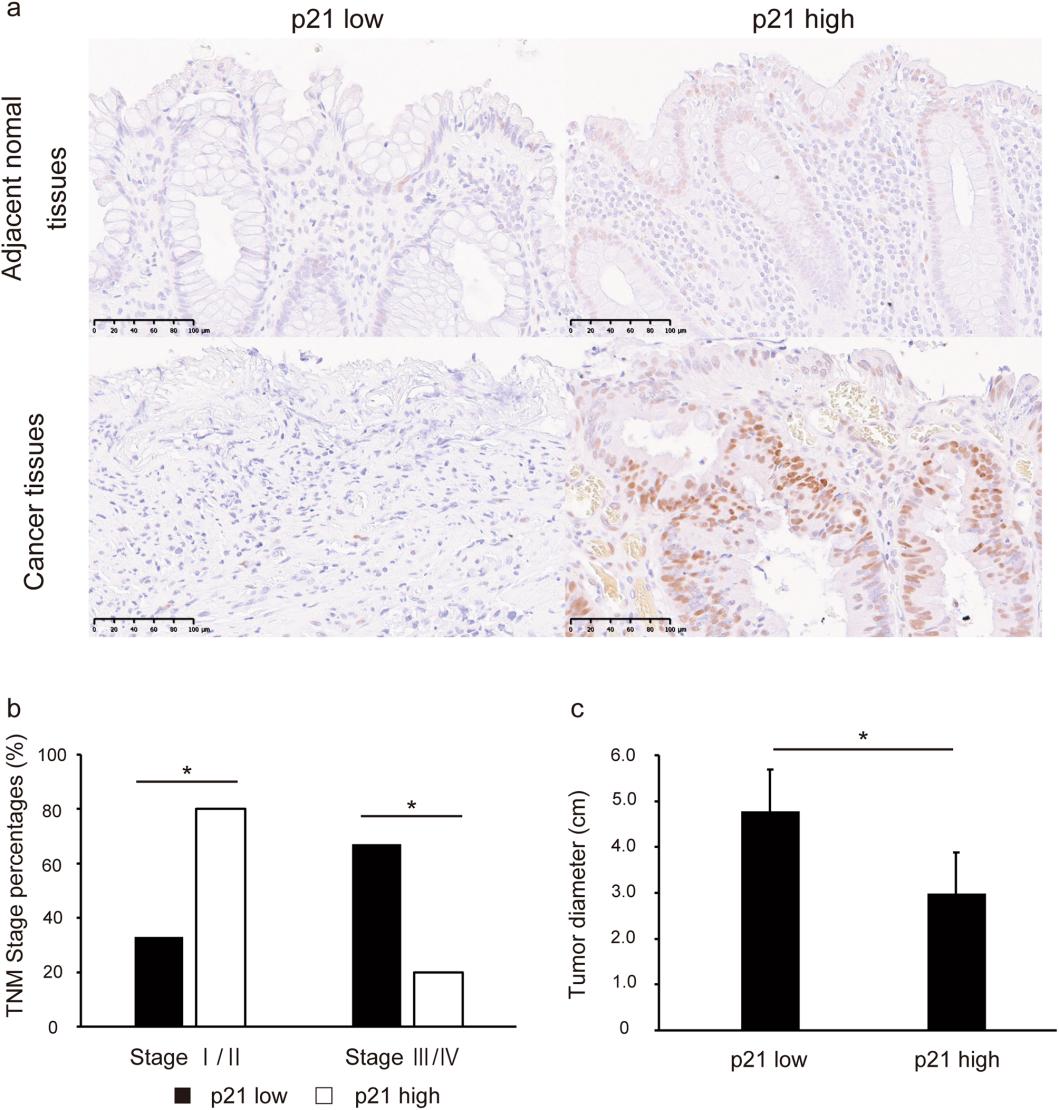
Low expression of p21 in advanced human CRC tissues. (**a)** Evaluation of p21 expression in human CRC tissues using immunohistochemical analysis. Data were generated from two representative CRC tissues for low and high p21 expression using the anti-p21 antibody. Scale bar: 100 μm. p21 expression in CRC tissue compared according to the TNM stage (**b**) and tumor size (**c**). Data are shown as mean ± standard error. *p < 0.05 compared to the high p21 expression group (Fisher’s exact test). *TNM* tumor, node, and metastasis, *CRC* colorectal cancer.

We analyzed tumor tissues from patients with CRC to investigate the role of p21 in CRC development in vivo. Fisher’s exact test was used to examine the correlation between p21 expression and clinicopathological characteristics. The clinicopathological characteristics of patients with CRC are summarized in Table 1. p21 expression was low in 55% (12/22) and high in 45% (10/22) of the CRC tissues (Fig. 6a). Immunohistochemical (IHC) analysis revealed that low p21 expression positively correlated with the tumor, node, metastasis stage (p = 0.043) and tumor diameter (p = 0.048) (Fig. 6b, c). In contrast, there was no association between p21 expression and age, sex, or tumor location. These results suggest that p21 expression is associated with CRC development.

## Discussion

This study investigated the effects of the PKR inhibitor C16 on CRC cells. C16 increased p21 expression and p21 upregulation induced G1 arrest and suppressed CRC cell proliferation.

Although there are several reports on the therapeutic effects of PKR inhibitors in neurological diseases^20–22^, there are only a few reports on the effects of PKR inhibitors on malignant tumors. Our previous study showed that C16 treatment suppresses tumor progression by suppressing the proliferation of tumor cells and the expression of angiogenesis-related growth factors, such as vascular endothelial growth factor, platelet-derived growth factor, fibroblast growth factors, and epidermal growth factor in HCC^19^. Furthermore, C16 induces apoptosis in a dose-dependent manner and suppresses CRC cell proliferation^23^, decreases p-eIF2α level, and induces MYC expression in patient-derived CRC organoid lines. However, previous studies did not demonstrate that C16 inhibits CRC cell proliferation via the cell cycle. In addition, MYC overexpression induces the progression of cell cycle^24,25^ and inhibits the induction of p21 expression^26,27^. Our results reveal that different pathways are involved in the effects of C16 treatment. To our knowledge, this is the first study to show that the inhibitory effect of C16 treatment on tumor growth is regulated by the CRC cell cycle.

We found that C16 suppressed the proliferation in the wild-type p53 CRC cells HCT116 and p53-mutated CRC cells HT29. Furthermore, C16 regulated the expression of cell cycle-related genes and increased p21 protein and mRNA expression independent of TP53 mutations. The western blot analysis showed that C16 did not affect the protein expression of p53 or phosphorylated p53, whereas the RT-PCR analysis showed that C16 increased the mRNA expression of *TP53* in HCT116 cells (wild-type TP53) but did not affect the mRNA expression of TP53 in HT29 cells (mutant type of TP53). Furthermore, the enrichment analysis showed that C16 affected the expression of cell cycle-related genes including TGF-β-related genes. Together, these findings suggest that an alternative pathway (such as the TGF-β pathway rather than the p53 pathway) is the key pathway by which C16 regulates the cell cycle.

ATP competitive inhibitors such as C16 have been reported to induce integrated stress responses depending on their concentration^28^. We conducted this study with C16 at a low concentration that is less likely to induce integrated stress responses (ISR). Furthermore, PKR inhibition by PKR knockdown suppressed cell proliferation, and cell proliferation inhibition by C16 was negated by p21 inhibition. From these results, it cannot be ascertained that C16 induced ISR. Further investigation is needed to elucidate the relationship with ISR.

Our analysis of human CRC tissues showed that p21 expression is related to CRC development. The expression of p21 decreased as tumor tissue atypicality increased. The expression of *p21* mRNA is lower in CRC tissues than in normal colorectal tissues according to a public database. Furthermore, a previous IHC study showed that p21 expression is inversely related to Ki67 proliferation and that it could be related to p53 inactivation^29,30^. Loss of p21 is associated with a poor prognosis in CRC^31^. In vitro and in vivo experiments demonstrate that p21 plays a role in tumor growth suppression in CRC cells^9,32,33^. In addition, loss of *CDKN1A* (p21) is associated with consensus molecular subtype (CMS) 4 CRC, which has worse relapse-free and overall survival rates compared to CMS 1–3^34^. Therefore, C16 may improve cancer prognosis by restoring p21 expression.

Several studies have investigated the effects of drugs and chemicals on p21 expression in cancer cells. For example, histone deacetylase inhibitors (trichostatin A, bortezomib, or doxorubicin) enhance p21 expression and induce cell cycle arrest^35,36^, demonstrating the tumor-suppressive effects of p21. However, there are contradictory reports on the tumor growth effects of p21^37,38^. p21 loss in colon cancer is associated with shorter survival among patients aged < 60 years, whereas it is associated with longer survival among patients aged ≥ 60 years^39^. p21 plays tumor-suppressive and tumor-proliferative roles; however, the development of clinically available p21-targeted therapeutics for CRC has not yet been achieved.

In conclusion, we found that C16 treatment inhibits tumor growth by regulating CRC cell cycle. Thus, PKR inhibition could be a novel p21-targeting therapy to regulate CRC.

## Methods

### Cell lines and cell culture

Human CRC cell lines HCT116 and HT29 were purchased from KAC (Kyoto, Japan). HCT116 and HT29 cells were cultured in McCoy’s 5A medium supplemented with 10% fetal bovine serum (Thermo Fisher Scientific, Waltham, MA, USA) and 1% penicillin. The cells were maintained at 37 °C in a humidified atmosphere of 5% CO_2_ and 95% air, and the culture medium was changed three times per week.

### PKR plasmids

Plasmids carrying *PKR* genes were kindly provided by Dr. Michael Gale, Jr. (University of Washington, Seattle, WA, USA). The plasmid pOS8, which expresses β-galactosidase, was used as the control plasmid. Each plasmid (1 mg/mL) was transfected into HCT116 at 50% confluence in six-well plates using Lipofectamine LTX with Plus Reagent (Thermo Fisher Scientific).

### RNA interference

We used PKR-specific siRNA1 (CAA AUU AGC UGU UGA GAU A, D-003527-01-0020), siRNA2 (GCA GAU ACA UCA GAG AUA A, D-003527-03-0020), and control siRNA (Dharmacon, Cambridge, UK). HCT116 cells were transfected with 50 pmol/L siRNA using RNAiMAX (Thermo Fisher Scientific). Twenty-four hours after transfection, protein was extracted from these cells.

### Chemicals

C16 was purchased from Merck (Darmstadt, Germany) and solubilized in dimethyl sulfoxide (DMSO). The final concentrations of PKR inhibitors used in the in vitro experiments are described in the following sections.

### Western blotting

RIPA buffer (50 mM HEPES KOH pH 7.9, 150 mM NaCl, 1.5 mM MgCl_2_, and 1.0% v/v NP-40) was added to the cells or tumors for protein extraction. Subsequently, 20 μg of protein was separated on 4–12% Bis–Tris gels (Thermo Fisher Scientific) and transferred onto an Immun-Blot PVDF Membrane for Protein Blotting (Bio-Rad Laboratories, Hercules, CA, USA). Alternatively, the separation was performed using the Jess system (automated simple western blot system; ProteinSimple, San Jose, CA, United States), with a 12–230 kDa separation module (#SM-W-004). The membranes were incubated with the following primary antibodies: anti-beta-actin (product number A5441) (Merck) and anti-p53 (sc-126) (Santa Cruz Biotechnology, Dallas, TX, USA). Antibodies against PKR (3210), p-p53 (Ser15) (9284), p-p53 (Ser392) (9281), and p21 (2947) were purchased from Cell Signaling Technology (Danvers, MA, USA). Anti-phosphorylated PKR (ab32036) antibody was purchased from Abcam (Cambridge, UK). Species-specific secondary antibodies, anti-rabbit (NA934) and anti-mouse (NXA931) antibodies, were purchased from Cytiva (Tokyo, Japan). Labeling was performed using an ECL prime kit (Cytiva) and visualized with ImageQuant LAS 4000 (Cytiva). The density of the bands was quantified via normalization to β-actin expression using the ImageJ software (National Institutes of Health, Bethesda, MD, USA). Data analysis was performed using the Compass software for Simple Western (version: 4.1.0).

### Cell proliferation assay

In vitro cell viability was quantified using the MTS assay (Promega, Fitchburg, WI, USA). After 24 h of siRNA or plasmid transfection in six-well plates, the cells were resuspended and seeded at a density of 2 × 10^3^ in 96-well plates. HCT116 and HT29 cells treated with C16 (Merck) at various concentrations (100–2000 nM) were seeded in 96-well plates at 2 × 10^3^ cells per well. The cells were treated with MTS reagent and incubated for 120 min. Absorbance was recorded at 450 nm. Cells treated with DMSO were used as controls. The wells were photographed under a microscope (ZEISS Axio Vert.A1; Carl Zeiss, Oberkochen, Germany).

### RNA-Seq and data analysis

Total RNA was collected from HCT116 cells 24 h after treatment with control DMSO or C16 diluted to 500 nM, and the integrity of the isolated RNA was verified using an Agilent 2100 Bioanalyzer (Agilent Technologies, Santa Clara, CA, USA). RNA samples with RNA integrity number > 9 were normalized to 100 ng/μL before further analysis. RNA-Seq libraries were prepared using the NEBNext Ultra II Directional RNA Library Prep Kit for Illumina (New England BioLabs; NEB, Ipswich, MA, USA), NEBNext Poly(A) mRNA Magnetic Isolation Module (NEB), and NEBNext Multiplex Oligos for Illumina (NEB) according to the manufacturer’s instructions and subsequently validated to ensure an average size of approximately 380–390 bp using the Agilent 2100 Bioanalyzer and the Agilent DNA1000 Kit (Agilent Technologies). Paired-end reads (75 bp) were sequenced using the MiSeq Reagent Kit V3 (Illumina, San Diego, CA, USA) on a MiSeq system (Illumina). Mapping to human genome data of hg38 was performed using Tophat (https://ccb.jhu.edu/software/tophat/index.shtml), and the expression analysis tool Cufflinks (http://cole-trapnell-lab.github.io/cufflinks/) was used to normalize expression in each sample before determining the differences in expression between the control DMSO (n = 3) and C16 (n = 3). Pathway and process enrichment analyses were performed using all genes after alignment and normalization with adjusted p < 0.05 using the Metascape and DAVID analysis tools^40,41^.

### Cell cycle analysis

HCT116 and HT29 cells were seeded at a density of 1 × 10^6^ cells/well in 10-cm dishes. A BD Cycletest™ Plus DNA Kit (BD Biosciences, Franklin Lakes, NJ, USA) was used to purify the samples at 24 h after treatment with C16 diluted to 500 nM, according to the manufacturer’s instructions, and cell cycle distribution was evaluated using flow cytometry (Gallios Flow Cytometer; Beckman Coulter, Brea, CA, USA).

### Apoptosis assay

HCT116 cells were treated with control DMSO or C16 diluted to 500 nM for 48 h. The attached cells were collected and assayed to evaluate apoptosis with annexin V-PE and 7-amino-actinomycin D (Tonbo Biosciences, San Diego, CA). To visualize stained cells, we used a FACSCalibur system (Becton Dickinson, Franklin Lakes, NJ). The data were analyzed with the FlowJo software (TreeStar Corporation, Ashland, OR).

### RNA extraction, cDNA synthesis, and real-time RT-PCR

Total RNA was extracted from HCT116 and HT29 cells using a RNeasy Plus Mini Kit (Qiagen, Venlo, Netherlands). Reverse transcription was performed using the SuperScript VILO™ cDNA Synthesis Kit (Thermo Fisher Scientific). Polymerase chain reaction amplification was performed using the following primers: *CDKN1A* forward primer (5′-CCGGCTGATCTT CTCCAAGAG-3′) and reverse primer (5′-AAGATGTAGAGCGGGCCTTTG-3′), *TP53* forward primer (5′-ATCCTCACCATCATCACACTGG-3′) and reverse primer (5′-ACAAACACGCACCTCAAAGC-3′), and *TBP* forward primer (5′-TGCTGCGGT AATCATGAGGATA-3′) and reverse primer (5′-TGAAGTCCAAGAACTTAGCTG GAA-3′) (Thermo Fisher Scientific). Real-time PCR was performed using a Light Cycler LC480 (Roche Diagnostics, Rotkreuz, Switzerland) with SYBR Green I dye (Roche) and TB Green Premix Ex Taq II (Takara Bio, Shiga, Japan) according to the manufacturer’s instructions. A constant amount of RNA was reverse transcribed to complementary DNA, and the complementary DNA was then amplified using the following conditions: 1 cycle of denaturation at 95 °C for 5 min, and 48 cycles of denaturation at 95 °C for 10 s, annealing at 60 °C for 5 s, and extension at 72 °C for 3 s. The expression of each gene was normalized to *TBP* mRNA level and is represented as fold changes compared to the control group.

### Patients and human colorectal tumor specimens

Human colorectal tumor samples were obtained from patients who underwent endoscopic submucosal dissection and surgery at our hospital between 2019 and 2020. The records of 25 cases with consecutive operative dates were examined, excluding 3 cases, death from another disease (n = 1) and follow-up at another hospital (n = 2). The clinical features of the 22 specimens are presented in Table 1.

### Ethics statement

Human sample analysis was approved by the Institutional Review Board (approval ID: 2206001) of Ehime University Hospital and performed in accordance with the ethical principles of the 1964 Declaration of Helsinki and its later amendments. Informed consent was obtained in the form of opt-out.

### Immunohistochemistry of CRC specimens

Colorectal tissues were fixed in formalin, and sections (3-μm thick) were cut from each block and stained using IHC. Paraffin-embedded samples were dewaxed and rehydrated, and antigen retrieval was performed by autoclaving for 1 min at 110 °C in citrate buffer (pH 6.0). Endogenous peroxidase activity was inactivated by incubation in methanol containing 1% hydrogen peroxidase for 20 min. The sections were subsequently incubated with 1% blocking goat serum for 30 min to reduce nonspecific reactions. For IHC, the sections were probed overnight with anti-p53 and anti-p21 antibodies (1:50), and incubated with a peroxidase-conjugated secondary antibody (Histofine Simple Stain Max POR; Nichirei, Tokyo, Japan) for 1 h at room temperature and then with Simple Stain DAB solution (Nichirei). Photomicrographs were captured using a Nikon ECLIPSE 50i microscope equipped with a digital camera (DS-F11; Nikon, Tokyo, Japan). The immunoreactivity was blindly assessed by two independent observers. p21 expression was classified as low (p21 immunostaining in less than 10% of the total tumor) or high (p21 immunostaining in more than 10% of the total tumor).

### Statistical analysis

All statistical analyses were performed using JMP software (v.17.0.0; SAS Institute, Cary, NC, USA). Data are expressed as mean and standard deviation. Differences were analyzed using the Mann–Whitney U test and Fisher’s exact test, and statistical significance was defined as p < 0.05 based on two-tailed tests.

### Supplementary Information

Supplementary Information.

## Data Availability

The datasets generated and/or analyzed during this study are available from the corresponding author on reasonable request.
