# Supplementary material for: C16, a PKR inhibitor, suppresses cell proliferation by regulating the cell cycle via p21 in colorectal cancer
Source: Sci Rep. 2024 Apr 19;14:9029. doi: 10.1038/s41598-024-59671-7 (PMC11031597; doi:10.1038/s41598-024-59671-7)
Supplement: Supplementary file 1 — Supplementary Information. [file 41598_2024_59671_MOESM1_ESM.pdf]

Supplementary Infomation

PKR inhibitor C16 treatment effects with regulated cell cycle in colorectal cancers

Yu Hashimoto, Yoshio Tokumoto, Takao Watanabe, Yusuke Ogi, Hiroki Sugishita, Satoshi Akita, Kazuki Niida, Mirai Hayashi, Masaya Okada, Kana Shiraishi, Kazuhiro Tange, Hideomi Tomida, Yasunori Yamamoto, Eiji Takeshita, Yoshio Ikeda, Taro Oshikiri, Yoichi Hiasa

Supplemental Table 1. RNA expression differences between cells treated with control DMSO and PKR inhibitor in cell cycle related gene

Reference	Gene Symbol	log2 (PKR inhibitor / DMSO)	p_value
NM_006142.5	SFN	1.11515	5.00E-05
NM_000660.7	TGFB1	0.91651	5.00E-05
NM_078467.3	CDKN1A	0.867238	5.00E-05
NM_057749.3	CCNE2	0.800557	0.0003
NM_001287604.2	ZBTB17	0.75717	5.00E-05
NM_001322262.2	CCNE1	0.671848	5.00E-05
NM_015391.4	ANAPC13	0.631214	0.0004
NM_001424057.1	CCND3	0.626335	5.00E-05
NM_001407013.1	SMAD3	-1.07494	5.00E-05
NM_001255.3	CDC20	-0.81272	5.00E-05
NM_004091.4	E2F2	-0.683678	5.00E-05
NM_001287518.2	CDC25B	-0.661611	5.00E-05
XM_017010635.3	ORC3	-0.596526	5.00E-05
NM_015675.4	GADD45B	-0.584855	0.00665
NM_004153.4	ORC1	-0.558552	0.00015
NM_001323281.2	RBL1	-0.541182	0.0049
NM_001143976.2	WEE1	-0.531096	5.00E-05
NM_006265.3	RAD21	-0.511548	0.0001
NM_012291.5	ESPL1	-0.47253	0.00055
NM_004219.4	PTTG1	-0.461726	0.00045
NM_004064.5	CDKN1B	-0.448763	0.0068
NM_001145306.2	CDK6	-0.442765	0.00055
NM_001166691.2	TTK	-0.422505	0.00185
NM_001362843.2	EP300	-0.416107	0.00245
NM_182776.3	MCM7	-0.403305	0.00335
NM_001278617.2	BUB1	-0.372097	0.0041
NM_005445.4	SMC3	-0.347959	0.0054
NM_005915.6	MCM6	-0.343461	0.0071

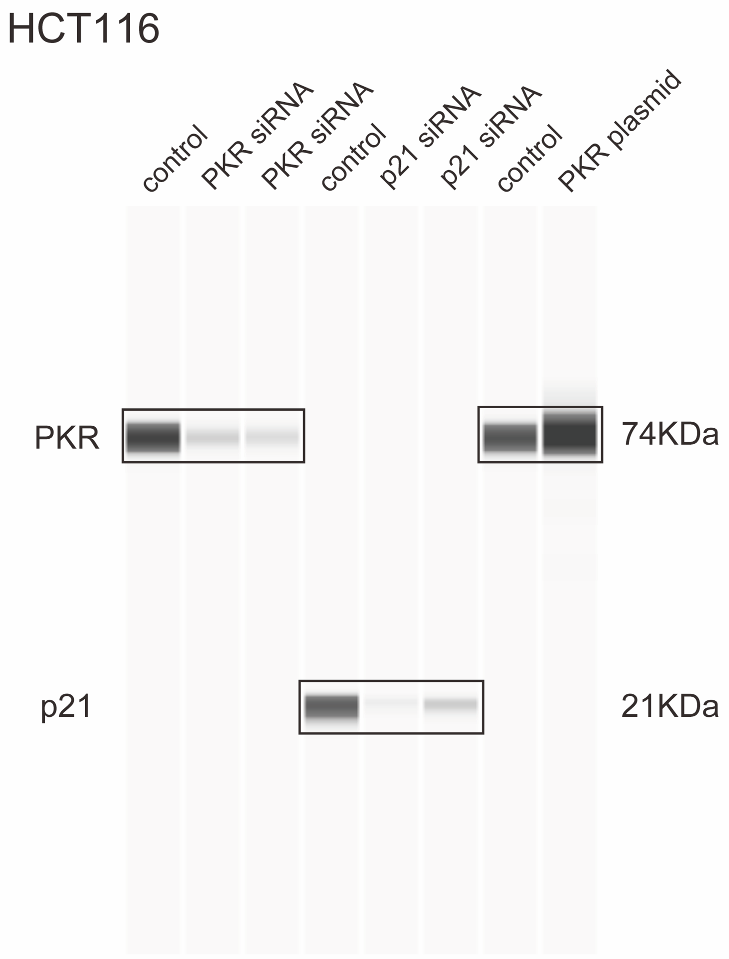
HCT116
control
PKR siRNA
PKR siRNA
control
p21 siRNA
p21 siRNA
control
PKR plasmid
PKR
74KDa
p21
21KDa

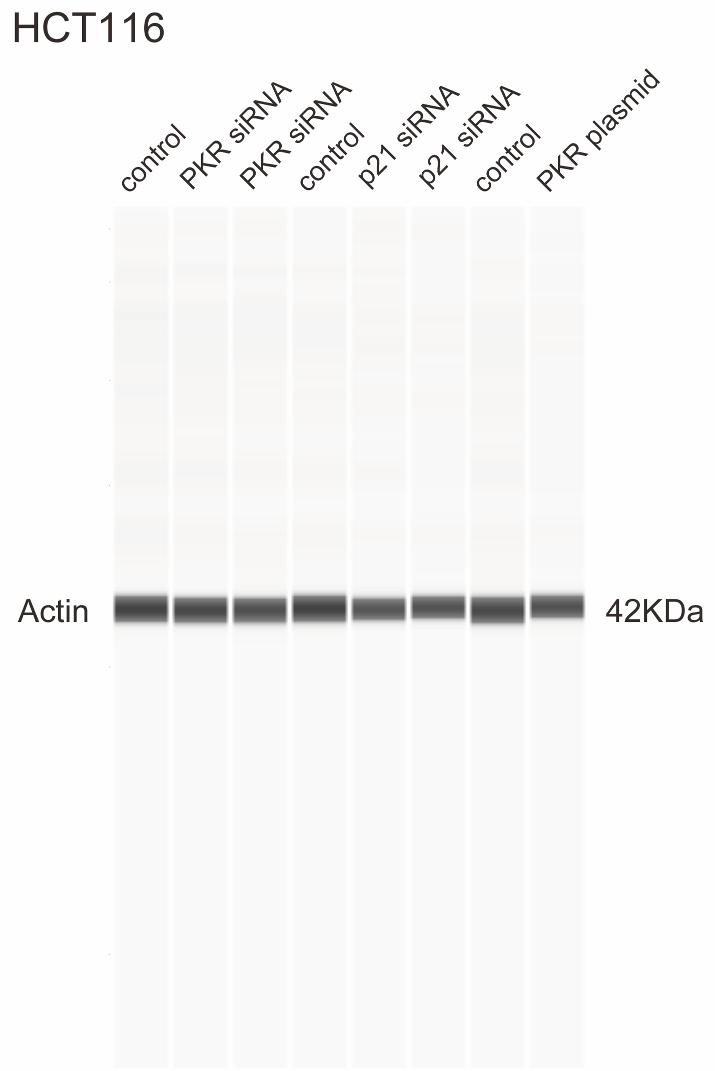

Supplementary Fig. S1. Original blots for Figure 1a, 5d

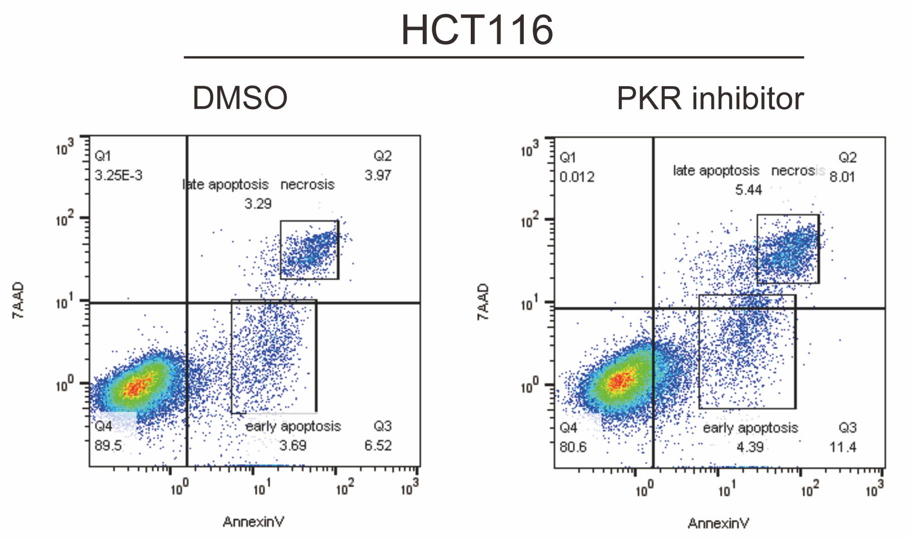

Supplementary Fig. S2. C16 leads to apoptosis in HCT116 cells. Flow cytometry analysis was performed after treatment with control DMSO or C16 diluted to 500 nM for 48h. Apoptotic cells were positive for annexin V, and necrotic cells were positive for 7-amino-actinomycin D.

HCT116

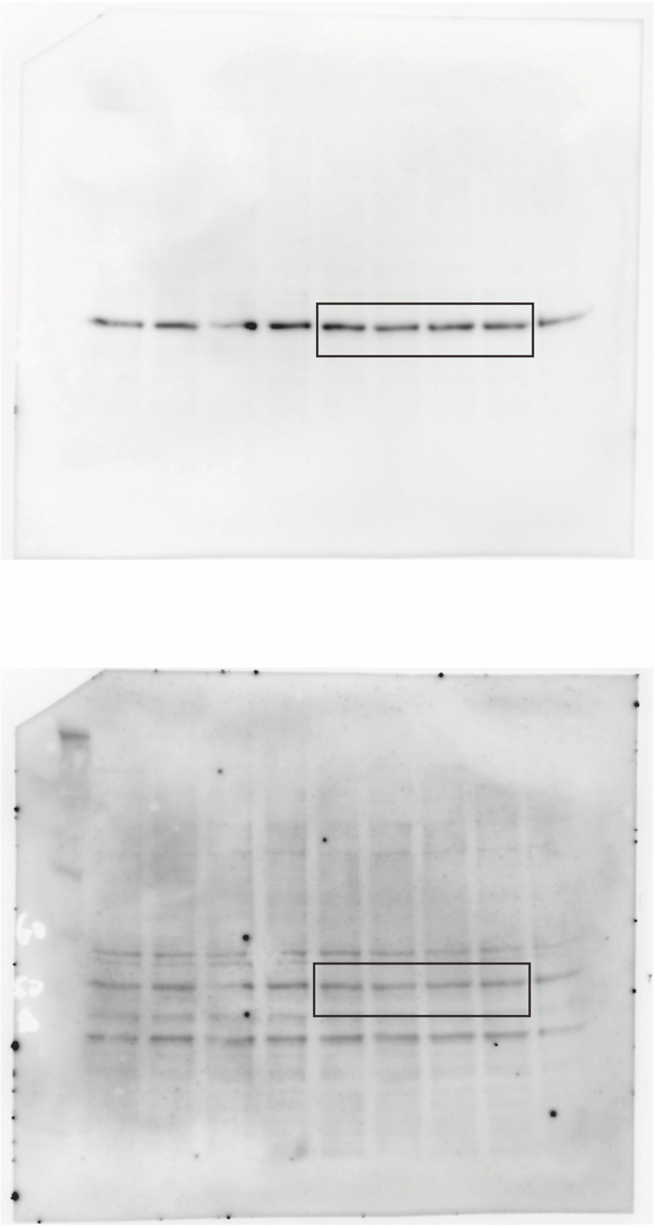

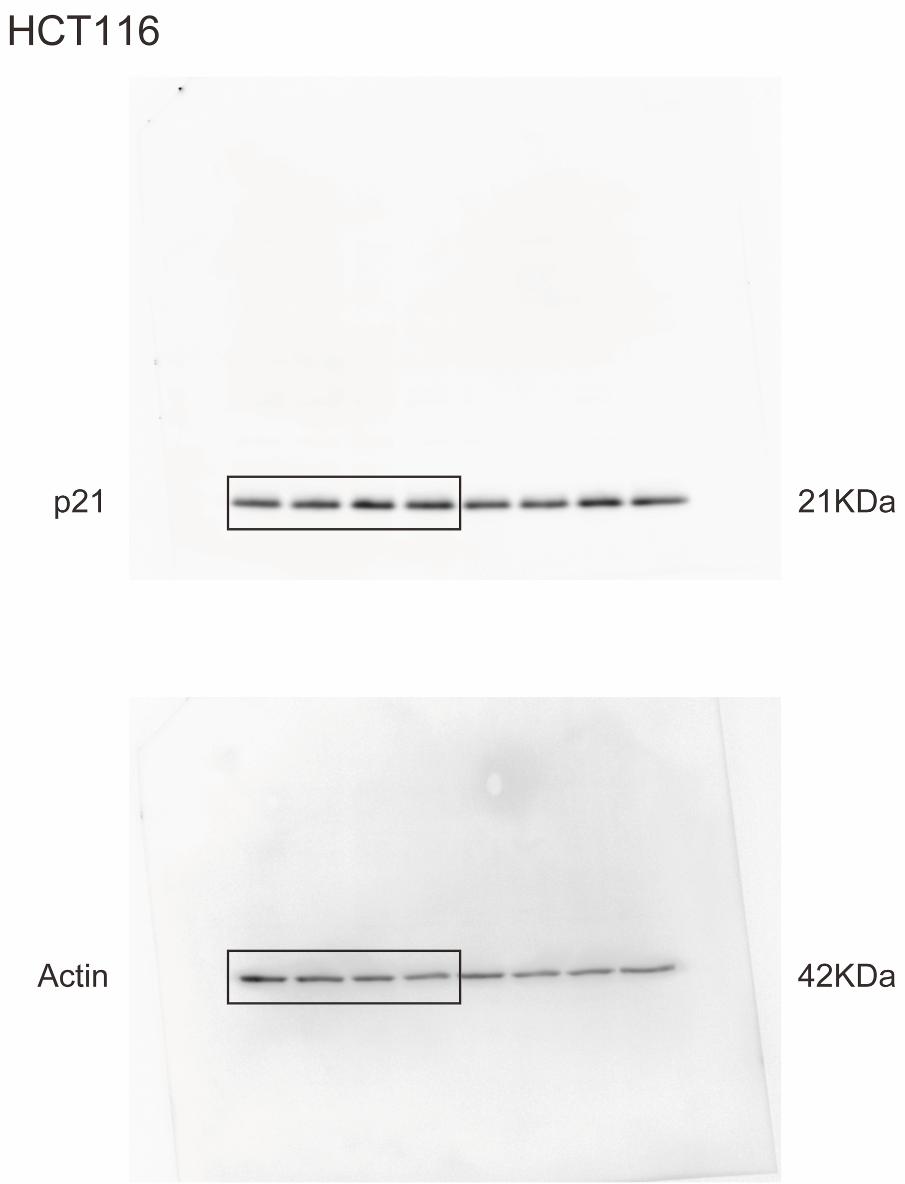
HCT116
p21
21KDa
Actin
42KDa

HT29

p53

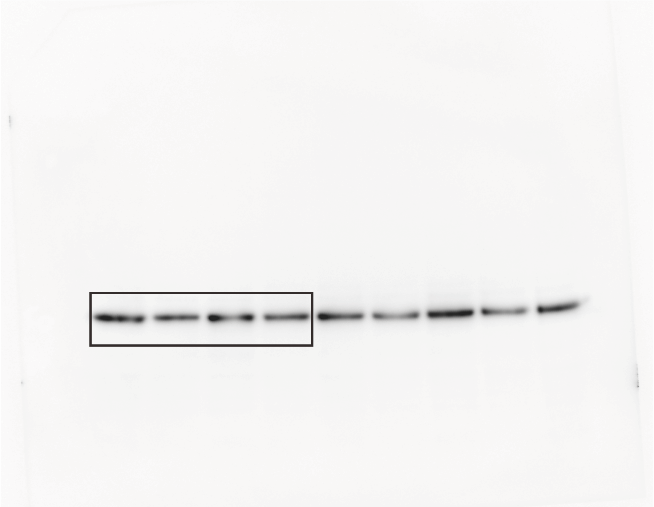

53KDa

phosphorylated
p53

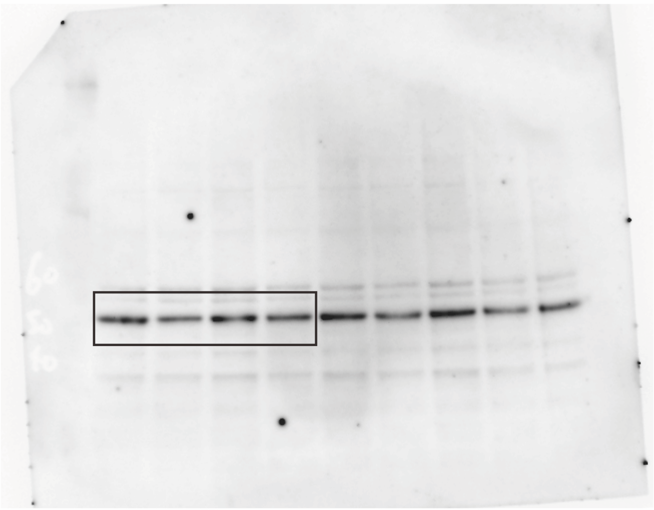

53KDa

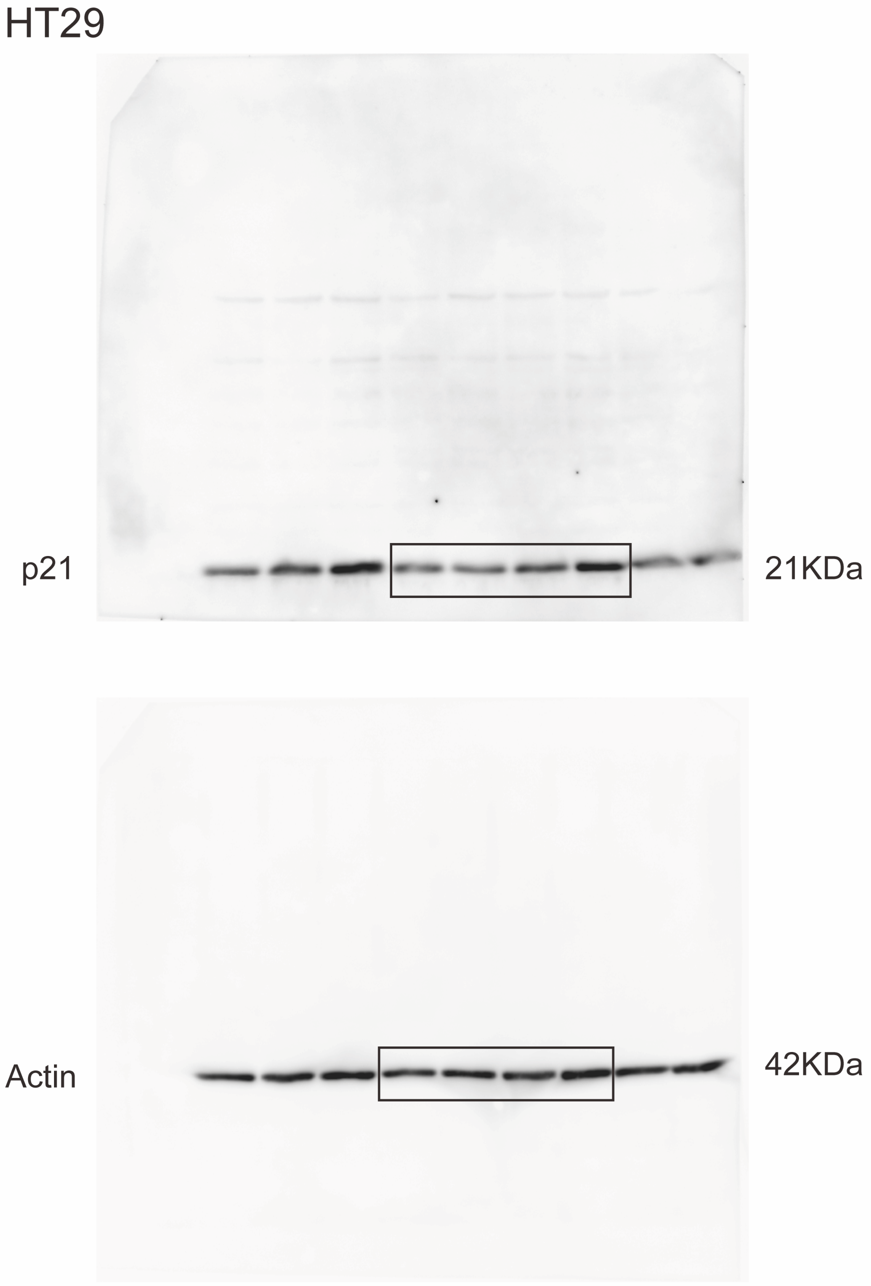

Supplementary Fig. S3. Original blots for Figure 5a
